# QRNet: A Quaternion-Based Retinex Framework for Enhanced Wireless Capsule Endoscopy Image Quality

**DOI:** 10.3390/bioengineering12030239

**Published:** 2025-02-26

**Authors:** Vladimir Frants, Sos Agaian

**Affiliations:** 1 Graduate Center, City University of New York, New York, NY 10016, USA; 2 Department of Computer Science, College of Staten Island, and the Graduate Center, The City University of New York, New York, NY 10314, USA; sos.agaian@csi.cuny.edu

**Keywords:** image processing, medical imaging, endoscopy, retinex, deep learning

## Abstract

Wireless capsule endoscopy (WCE) offers a non-invasive diagnostic alternative for the gastrointestinal tract using a battery-powered capsule. Despite advantages, WCE encounters issues with video quality and diagnostic accuracy, often resulting in missing rates of 1–20%. These challenges stem from weak texture characteristics due to non-Lambertian tissue reflections, uneven illumination, and the necessity of color fidelity. Traditional Retinex-based methods used for image enhancement are suboptimal for endoscopy, as they frequently compromise anatomical detail while distorting color. To address these limitations, we introduce QRNet, a novel quaternion-based Retinex framework. QRNet performs image decomposition into reflectance and illumination components within hypercomplex space, maintaining inter-channel relationships that preserve color fidelity. A quaternion wavelet attention mechanism refines essential features while suppressing noise, balancing enhancement and fidelity through an innovative loss function. Experiments on Kvasir-Capsule and Red Lesion Endoscopy datasets demonstrate notable improvements in metrics such as PSNR (+2.3 dB), SSIM (+0.089), and LPIPS (−0.126). Moreover, lesion segmentation accuracy increases by up to 5%, indicating the framework’s potential for improving early-stage lesion detection. Ablation studies highlight the quaternion representation’s pivotal role in maintaining color consistency, confirming the promise of this advanced approach for clinical settings.

## 1. Introduction

Gastrointestinal (GI) diseases pose a significant global health challenge, significantly affecting patient well-being and healthcare resources. In the United States, total annual expenditures for GI conditions reached USD 136.9 billion in 2015, exceeding the costs of many other common disorders [1]. In addition to direct healthcare costs, GI conditions incur considerable indirect expenses, including reduced work productivity. Irritable bowel syndrome (IBS) alone has been linked to up to USD 20 billion in annual indirect costs [2]. Colorectal cancer (CRC) represents a major concern within the GI field. Recent increases in CRC incidence among younger adults warrant special attention. The American Cancer Society reports that 20% of CRC diagnoses in 2019 occurred in individuals under age 55, nearly double the rate observed in 1995 [3].

Endoscopic evaluation, mainly through wireless capsule endoscopy (WCE), has revolutionized the detection of gastrointestinal (GI) abnormalities and colorectal cancer (CRC). While effective, traditional endoscopic methods challenge patient comfort and visualization of complex GI regions. WCE addresses these limitations through a miniature capsule (9 × 26 mm) equipped with advanced imaging capabilities [4,5]. However, technical constraints in illumination and camera performance often result in suboptimal image quality, potentially missing crucial diagnostic indicators. While recent diffusion-based deep learning approaches show promise in image enhancement, they often fall short in quantitative metrics and downstream tasks like lesion segmentation [6,7]. Nevertheless, traditional approaches for low-light enhancement do not take into account the specifics of WCE imaging and tend to produce subpar results with limited color fidelity [6,8,9]. While many general-purpose image enhancement algorithms prioritize overall image quality and noise suppression, medical imaging requires a careful balance between these improvements and the faithful representation of the color.

The unique physiological environment of endoscopic imaging presents distinct technical challenges. Non-Lambertian tissue reflections create weak texture characteristics, while point light sources result in inconsistent illumination. These obstacles and stringent color accuracy requirements render conventional enhancement methods inadequate [5,6]. Medical imaging necessitates a delicate balance between overall image quality and precise color representation since even minor variations in hue or saturation can significantly impact diagnostic accuracy [10]. Key challenges in WCE imaging include the following:

- Limited illumination in GI environments, resulting in underexposed anatomical features;
- Contrast problems from overexposure and reflective surfaces;
- Color distortion due to independent channel processing;
- Loss of inter-channel relationships critical for accurate diagnosis;
- The trade-off between enhancement and preservation of diagnostic features.

The Retinex theory, despite its success in modeling human color vision, exhibits limitations in endoscopic applications. Current implementations struggle to preserve texture details and color fidelity, which are crucial for accurate diagnosis. Traditional RGB channel processing methods often introduce artifacts and do not maintain essential inter-channel relationships for medical imaging.

The main contributions of this work can be summarized as follows:

1. A specialized mamba-based network architecture optimized for endoscopic environments reduces computational overhead through efficient parameter usage, enables real-time processing on resource-limited endoscopic devices, maintains high enhancement quality while using 30% fewer parameters than conventional architectures, and facilitates deployment in clinical settings where computing resources are constrained.
2. A novel Retinex framework for comprehensive RGB channel processing: Preserves crucial diagnostic details through quaternion-valued representation; enhances color accuracy by separately handling reflectance and illumination components; reduces color distortion common in traditional enhancement methods; and Improves visibility of subtle tissue variations and lesions through better color fidelity.
3. A complete framework for endoscopic image enhancement with a custom loss function delivers consistently superior results across multiple objective quality metrics; provides enhanced visual clarity that aids in detecting subtle pathological changes; Improves diagnostic accuracy by highlighting clinically relevant features; and maintains natural tissue appearance while enhancing the visibility of essential structures.
4. Extensive evaluation: extensive evaluation using the Kvasir-Capsule (2000 images) and Red Lesion Endoscopy (1500 images) datasets demonstrate significant improvements: PSNR improvement (+2.3 dB), SSIM enhancement (+0.089), LPIPS reduction (−0.126), and lesion segmentation accuracy (+5%). Ablation studies validate the effectiveness of the quaternion representation in color preservation, which is particularly beneficial for detecting early-stage lesions in challenging lighting conditions.

The remainder of this paper is organized as follows: Section 2 provides a brief overview of related work, Section 3 discusses the method used in our experiments, Section 4 presents and analyzes our results, Section 5 offers a broader discussion of the findings, and Section 6 concludes the study.

## 2. Literature Review

WCE has opened new avenues for medical diagnostics, providing a non-invasive way to visualize the GI tract. By integrating capsule cameras with state-of-the-art computer vision and image processing algorithms, clinicians can detect and characterize various GI pathologies more effectively. For instance, Nam et al. utilized stereo camera-based capsule endoscopy for three-dimensional (3D) reconstruction of small bowel lesions in patients presenting with gastrointestinal bleeding, Crohn’s disease, tumors, and unexplained abdominal pain [11]. Tanwar et al. employed convolutional neural networks (CNNs) with guided image filtering and histogram equalization to enhance colonoscopic images, leading to a more accurate classification of colorectal polyps [12]. Stoleru et al. leveraged endoscopic images to develop machine learning algorithms for automated celiac disease detection, demonstrating improved diagnostic accuracy [13]. Beyond direct visualization, 3D reconstruction and simultaneous localization and mapping (SLAM) techniques have been adopted to support surgical navigation and planning in endoscopic settings [14].

Despite these advancements, low-light imaging remains a critical limitation for WCE. Restricted illumination within the GI tract makes it challenging to capture sufficient visual details, negatively affecting the performance of classical and deep learning-based algorithms. Traditional enhancement methods, such as gamma correction, histogram equalization, and traditional Retinex-based approaches, can be limited when dealing with extreme underexposure or complex noise patterns [15].

Retinex theory models human color perception and is the basis of a wide range of classical image enhancement approaches. It states that an image can be decomposed into two primary components: reflectance, corresponding to the intrinsic color of objects, and illumination, representing the lighting conditions under which the scene is captured. Enhancement typically involves adjusting the illumination component while preserving the reflectance. One of the earliest implementations, the Single-Scale Retinex (SSR), uses a center/surround mechanism to enhance local contrast. However, due to its simplistic single-scale design, SSR often introduces noise and color distortion [16]. The Multi-Scale Retinex (MSR) algorithm, proposed by Rahman et al., mitigates some of SSR’s drawbacks by incorporating multiple scales, enhancing images more comprehensively across different spatial frequencies [17]. Jobson et al. further extended MSR to include color recovery (MSRCR), reducing color distortions but sometimes introducing halo artifacts [18].

Subsequent improvements in Retinex-based methods emphasized more accurate separation of the reflectance and illumination components. Fu et al. introduced a weighted variational model (SRIE) to achieve simultaneous reflectance and illumination estimation, successfully suppressing artifacts associated with abrupt intensity changes [19]. Guo et al. proposed a structure-aware smoothing algorithm (LIME) that refines the estimated illumination map, preserving critical structural details in low-light areas [8]. Specifically for the endoscopy domain, Wang et al. incorporated the inverse square law of illumination into Retinex theory, demonstrating effective dynamic range compression for medical imaging [20].

Beyond the classical Retinex, variational models and fusion strategies have also gained popularity in low-light image enhancement. Ng and Wang applied total variation (TV) regularization to Retinex decomposition, achieving better noise suppression [21]. Nevertheless, strict regularization can cause over-smoothing of important edges, especially in darker regions. Fusion-based approaches, where multiple contrast-enhanced versions of the illumination map are merged, offer superior detail restoration but may inadvertently amplify noise in scenes with significant illumination disparities [22].

The advent of deep learning has extended Retinex-based enhancement, allowing end-to-end training. Wei et al. introduced RetinexNet, a two-stage network that first decomposes an image and then refines its illumination with a trainable module [9]. However, it sometimes intensifies noise in extremely dark conditions. Zhang et al. proposed KinD, which splits the enhancement process into layer decomposition, reflectance restoration, and illumination adjustment, effectively improving global and local consistency [23]. Other methods, such as URetinex-Net [24], RetinexDIP [25], and RRDNet [26], refine the decomposition process via deep unfolding, self-supervision, or zero-shot learning but can be computationally expensive or prone to color artifacts.

Recently, transformer architectures have been integrated with Retinex theory to handle complex noise and non-uniform lighting. Cai et al. developed RetinexFormer, a one-stage transformer-based framework for low-light enhancement, but it still treats reflectance and illumination sequentially [27]. Further studies incorporate diffusion models or more elaborate pipelines (e.g., RetinexMamba, Diff-Retinex), improving generative fidelity at the expense of high computational cost [28,29]. Moreover, these methods generally rely on scalar-valued operations, which can lose critical inter-channel correlations and introduce unnatural color shifts under severe low-light conditions [30,31,32].

While Retinex and deep-learning-based approaches are widely explored, in general, in low-light scenarios, the number of dedicated methods for endoscopic images is limited. Bai et al. introduced the LLCaps framework, combining a multi-scale CNN with a reverse diffusion process for refined reconstruction. Their Curved Wavelet Attention block captures subtle high-frequency details crucial for diagnostic accuracy, showcasing significant improvements in lesion segmentation on real WCE data [6]. Chen et al. proposed LighTDiff, a lightweight diffusion-based framework tailored for surgical endoscopy, employing a T-shaped model design to balance global structural capture with fine detail reconstruction. By integrating the Temporal Light Unit and a Chroma Balancer, they reduced color shifts and model size, making the approach more practical for resource-constrained environments [7].

Additional specialized research has targeted specific aspects of endoscopic imaging. Gómez et al. tackled low-light challenges in high-speed endoscopy using a CNN-based approach that generates artificial darkening through Perlin noise for data augmentation, leading to substantial quality improvements [33]. Xia et al. handled uneven illumination in stereoscopic endoscopic images by adaptively boosting dark regions, successfully reducing noise amplification [34]. Despite these advances, a universal solution that robustly preserves color fidelity, structural detail, and noise reduction under extreme low-light conditions, particularly for capsule-based devices, is still a challenge.

Classical Retinex can introduce artifacts and over-smooth details. Variational and fusion strategies often struggle with high noise or extreme illumination disparities, while deep learning solutions can be computationally heavy and susceptible to color inaccuracies. Moreover, WCE introduces domain-specific challenges such as size constraints, uneven illumination, and minimal color distortion, requiring tailored algorithms.

Motivated by these gaps, we aim to develop a quaternion-based Retinex framework specifically designed to enhance low-light capsule endoscopy images. Our approach seeks to incorporate multidimensional color information, maintain structural fidelity, and preserve hue accuracy, ultimately improving the diagnostic capabilities of WCE under challenging illumination conditions.

## 3. Materials and Methods

### 3.1. Learning in Decomposition Transform Domain

To address the complex interplay of low-light conditions, uneven illumination, and stringent color fidelity requirements in wireless capsule endoscopy (WCE), we use a Retinex decomposition that learns to separate each input image S into two quaternion-valued maps, $Q_{1}$ and $Q_{2}$. One map, $Q_{1}$, serves as a reflectance-like component capturing stable color and texture features, while the other map, $Q_{2}$, encapsulates illumination amplitudes. The decomposition is formulated via the Hamilton product:

$$\hat{S}\left(x,y\right)=Q_{1}\left(x,y\right)⊗Q_{2}\left(x,y\right)$$

where ⊗ denotes quaternion multiplication.

Each 3-channel pixel $\left(R\left(x,y\right),G\left(x,y\right),B\left(x,y\right)\right)$ is first encoded into a purely imaginary quaternion:

$$q_{\mathrm{color}}\left(x,y\right)=0+R\;i+G\;j+B\;k.$$

which preserves inter-channel dependencies essential for color constancy in diagnostic imagery. We initialize

$$Q_{1}^{\mathrm{init}}\left(x,y\right)=\frac{q_{\mathrm{color}}\left(x,y\right)}{q_{\max}\left(x,y\right)+\varepsilon },\;Q_{2}^{\mathrm{init}}\left(x,y\right)=q_{\mathrm{color}}\left(x,y\right)$$

where $q_{\max}\left(x,y\right)$ represents the maximum of $\left\{R,\;G,\;B\right\}$, and ε is a small constant. Thus, $Q_{1}^{\mathrm{init}}$ approximates a normalized “color only” component while $Q_{2}^{\mathrm{init}}$ contains the original magnitudes.

To exploit multi-scale spatial context, each quaternion channel $\left(r,i,j,k\right)$ in $Q_{1}$ and $Q_{2}$ is first wavelet-transformed using the Haar transform, yielding four sub-bands $\left\{T_{LL},T_{LH},T_{HL},T_{HH}\right\}$ for each channel. This separates low-frequency illumination details from high-frequency structural features. We then concatenate these sub-bands across the channel dimension, forming a compact tensor for processing in the wavelet domain.

### 3.2. Proposed Framework

**Figure 1:**
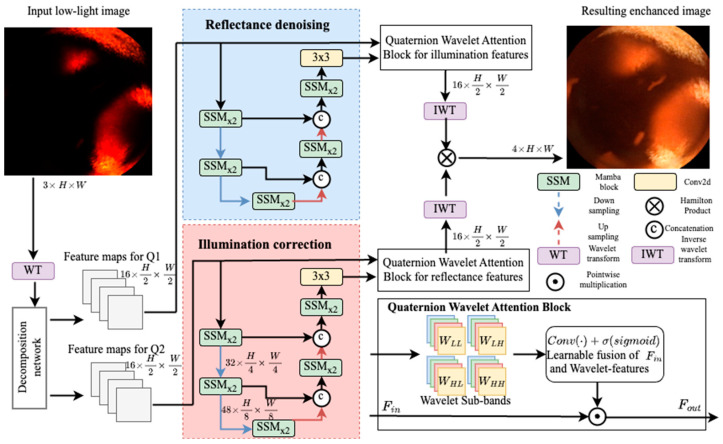
An overview of the proposed **QRNet** pipeline for low-light WCE enhancement. The input image is decomposed into two feature maps $Q_{1}$ (reflectance) and $Q_{2}$ (illumination), each processed by separate SSM Mamba-based encoder–decoder branches. Wavelet-transformed sub-bands guide an attention mechanism that refines both reflectance and illumination features. Finally, the enhanced quaternions are combined via the Hamilton product to generate the output image with improved contrast and preserved color fidelity.

Figure 1 Presents QRNet framework. First, a trainable decomposition network, a set of convolutional layers, generates estimations of $Q_{1}$ and $Q_{2}$ in wavelet transform domain. Afterward, an inverse wavelet transform reconstructs the refined quaternion channels to the original resolution. Decomposition part is trained separately on pairs of low-light and normal light using loss function from [9]. To ensure $Q_{1}$ captures stable color/texture (reflectance) and $Q_{2}$ encapsulates illumination, we initialize our decomposition by dividing each color channel by its maximum intensity, thus emphasizing texture and color invariance in $Q_{1}$. Simultaneously, the loss function enforces smooth illumination in $Q_{2}$ and preserves detailed reflectance in $Q_{1}$. Empirically, we observe $Q_{1}$ to be relatively invariant to global lighting shifts and more focused on structural details while $Q_{2}$ learns to represent the overall brightness amplitudes.

Once trained, the decomposition network’s weights are frozen, and the framework then applies two SSM Mamba-based encoder–decoder modules [35]. The first module, the reflectance denoiser, operates exclusively on $Q_{1}$, focusing on noise removal noise while carefully preserving texture details and maintaining the color fidelity of tissue structures. The second module, Illumination Corrector, operates on $Q_{2}$ to stabilize lighting amplitudes and reduce underexposure.

Within each encoder–decoder, hierarchical features are extracted at multiple scales using convolutional and self-attention layers. Skip connections facilitate the flow of spatial details, while Mamba blocks (Interleaved Group Attention modules [28]) further refine intermediate representations. By treating $Q_{1}$ and $Q_{2}$ separately, the network can more effectively tailor denoising and illumination correction to each component’s characteristics.

Empirical observations demonstrate that $Q_{1}$ exhibits strong invariance to global lighting changes while effectively capturing structural and textural details. Meanwhile, $Q_{2}$ successfully learns to represent the overall distribution of brightness. This two-stage training approach offers several key advantages: it ensures decomposition stability before enhancement, prevents interference between decomposition and enhancement learning, allows specialized optimization for each component, and maintains the integrity of reflectance and illumination information throughout the process.

Quaternion Wavelet Attention Block: A key element of the enhancement process is the wavelet-guided attention that leverages the previously computed wavelet features. The network reintroduces the sub-band coefficients (extracted in the decomposition stage) as an additional input to an attention mechanism. This encourages the model to highlight relevant for correct reflectance or illumination estimation. The attention module computes importance scores for different sub-bands and spatial regions. High-frequency features (e.g., edges, lesion boundaries) can be emphasized in $Q_{1}$ whereas low-frequency patches (overall brightness distribution) are reweighted in $Q_{2}$. The refined attention maps modulate each branch’s intermediate features, ensuring that final outputs retain crucial structural details and maintain consistent color without over-enhancement or banding artifacts.

A concise way to express the wavelet-guided attention operation is to view it as a learnable function that reweights (or “gates”) the main feature map $F_{in}$ by referencing the sub-band coefficients $\left\{\;W_{\mathrm{LL}},W_{\mathrm{LH}},W_{\mathrm{HL}},W_{\mathrm{HH}}\;\right\}$:

$$F_{out}=F_{in}⊙\sigma (Conv\left(Concat\left(F_{in};W_{\mathrm{LL}};W_{\mathrm{LH}};W_{\mathrm{HL}};W_{\mathrm{HH}}\right)\right))$$

where

- $F_{in}$ is the current feature map from either the reflectance branch $Q_{1}$ or the illumination branch $Q_{2}$.
- $\left\{\;W_{\mathrm{LL}},W_{\mathrm{LH}},W_{\mathrm{HL}},W_{\mathrm{HH}}\;\right\}$ are the Haar wavelet sub-band features computed in the decomposition stage.
- Conv(⋅) is a learnable convolution that fuses these inputs to produce attention weights.
- σ(⋅) is a sigmoid (or similar) activation that normalizes the weights.
- ⊙ denotes elementwise multiplication.

This gating mechanism highlights high-frequency edges or texture in $Q_{1}$ and modulates low-frequency brightness patterns in $Q_{2}$, thus helping preserve fine details while ensuring coherent illumination.

Once the reflectance and illumination encoder–decoder streams have produced enhanced $Q_{1}$ and $Q_{2}$, the two quaternions are recombined via the Hamilton product:

$$Q_{out}=Q_{1}^{enh}⊗Q_{2}^{enh}$$

The final enhanced RGB image $\hat{S}$ is extracted from the imaginary parts $\left\{i,j,k\right\}$ of $Q_{out}$. The proposed approach can significantly improve low-light WCE images while preserving the subtle color variations critical for reliable lesion detection by explicitly modeling reflectance and illumination in hypercomplex space and integrating wavelet-driven attention.

### 3.3. Loss Function

Accurate preservation of both structural detail and color fidelity is imperative in WCE. To this end, we use a multi-term loss function L that combines reconstruction, perceptual, and frequency-based objectives:

$$L=\lambda_{pix}L_{pix}+\lambda_{percep}L_{percep}+\lambda_{fft}L_{fft}+\lambda_{ssim}L_{ssim}$$

where

- $\lambda_{pix}$ is $l_{1}$ pixel-wise reconstruction loss directly comparing $\hat{S}$ and the ground truth $S_{gt}$.
- $L_{percep}$ uses pretrained feature networks (VGG16 encoder) to encourage perceptual similarity in high-level representations [36].
- $L_{percep}$ is a frequency-domain loss computed by taking the 2D FFT of both $\hat{S}$ and $S_{gt}$ measuring their discrepancy in the log-magnitude space [37]. This helps mitigate color banding and enforces consistent global illumination.
- $L_{ssim}$ enforces structural similarity at a global scale [38].

Empirically, combining these terms ensures that the network (i) recovers fine anatomical detail in underexposed regions, (ii) avoids color shifts detrimental to diagnosis, and (iii) enhances illumination consistency without degrading overall contrast. In our experiments, we use $\lambda_{pix}=1.0$, $\lambda_{percep}=0.05$, $\lambda_{fft}=0.05$, $\lambda_{ssim}=0.20$.

## 4. Results

In this section, we present our experimental findings on low-light WCE image enhancement and subsequent lesion segmentation. We begin by describing the datasets used (Section 4.1) and introducing the evaluation metrics (Section 4.2). We then summarize our implementation details and the competing methods (Section 4.3). Finally, we discuss our quantitative and qualitative results for low-light enhancement on two public datasets (Section 4.4) and report the segmentation outcomes using TransUNet (Section 4.5).

### 4.1. Dataset

For experiments, we adapt synthetic datasets presented in [6].

The Kvasir-Capsule (KC) dataset is publicly available and includes both anatomical categories and various luminal findings [39]. A total of 2400 images are randomly selected, with 2000 used for training and 400 for testing. A data augmentation strategy is implemented to simulate low-light scenarios involving random gamma corrections and overall brightness reductions. Additionally, a separate external validation set of 100 images, naturally low in brightness from the Kvasir-Capsule, is extracted to further assess the model’s generalizability under real low-light conditions.

Red Lesion Endoscopy (RLE) dataset focuses on red lesion segmentation tasks, such as angioectasias and bleeding [40]. A total of 1283 images were randomly selected, with 946 used for training and 337 for testing. The same low-light augmentation procedure as in the Kvasir Capsule is applied. The dataset includes segmentation masks to conduct segmentation experiments on these RLE images, evaluating the clinical relevance of image enhancement approaches.

### 4.2. Metrics

We employ three standard metrics for image enhancement quality:

Peak Signal-to-Noise Ratio (PSNR) [41]:

$$PSNR=10{log}_{10}\left(\frac{{MAX}^{2}}{MSE}\right)$$

where MAX is the maximum possible intensity (e.g., 255 for 8-bit images), and MSE is the mean squared error between the enhanced and ground-truth images. Higher PSNR indicates lower distortion.

Structural Similarity Index (SSIM) [42]:

$$SSIM\left(x,y\right)=\frac{\left(2\mu_{x}\mu_{y}+c_{1}\right)\left(2\sigma_{xy}+c_{2}\right)}{\left(\mu_{x}^{2}+\mu_{y}^{2}+c_{1}\right)\left(\sigma_{x}^{2}+\sigma_{y}^{2}+c_{2}\right)}$$

where $\mu_{x}$ and $\mu_{y}$ are mean intensities, $\sigma_{x}^{2}$ and $\sigma_{y}^{2}$ are variances, and $\sigma_{xy}$ is the convariance of the two images. $c_{1}$ and $c_{2}$ are stability constants. SSIM gauges structural fidelity; higher is better.

Learned Perceptual Image Patch Similarity (LPIPS) [43]:

LPIPS measures perceptual distance by comparing deep features extracted from a pretrained network. Lower LPIPS values denote closer perceptual similarity to the reference.

Additionally, we report a no-reference perceptual metric NIQE (Naturalness Image Quality Evaluator), which is a lower-is-better measure for overall quality [44,45]. Although less commonly used in medical images, they provide an auxiliary perspective on perceptual fidelity.

For segmentation, we use standard metrics such as f1-score, mean Intersection over Union (mIoU), accuracy, precision, sensitivity, and specificity to quantify performance on lesion detection [46].

### 4.3. Implementation Details and Methods for Comparison

All methods, including our quaternion QRNet approach, are trained from scratch with a learning rate of $10^{-4}$ for 200 epochs on both the Kvasir-Capsule and RLE training splits. We compare the following against eight state-of-the-art methods:

1. LIME [8]: A classic low-light enhancement method using illumination maps.
2. ZeroDCE [47]: A zero-reference deep curve estimation technique for low-light adjustment.
3. LLFormer [48]: A transformer-based model specialized in low-light image correction.
4. LytNet [31]: A lightweight CNN framework aiming for real-time low-light enhancement.
5. WaveNet [49]: Uses wavelet transforms to enhance image details in the frequency domain.
6. HVI-CIDNet [32]: A hybrid visual intelligence model for contrast-illumination-denoising.
7. RetinexFormer [27]: A Transformer variant that decomposes images via a Retinex-inspired design.
8. LightTDiff [7]: A diffusion-based approach that iteratively refines the image, often expensive computationally.

### 4.4. Low-Light Image Enhancement Results

Table 1 compares each method on the Kvasir-Capsule dataset set using PSNR, SSIM, and LPIPS. Our approach achieves the highest PSNR (37.781 dB) and SSIM (0.976) alongside the lowest LPIPS (0.022). Notably, LightTDiff, despite being a diffusion model with strong visual results, yields a lower PSNR (31.413 dB) and a higher LPIPS (0.067). Traditional methods like LIME and ZeroDCE significantly underperform in both PSNR and SSIM, indicating that their simplistic adjustments introduce color shifts and fail to restore structural information.

**Figure 2:**
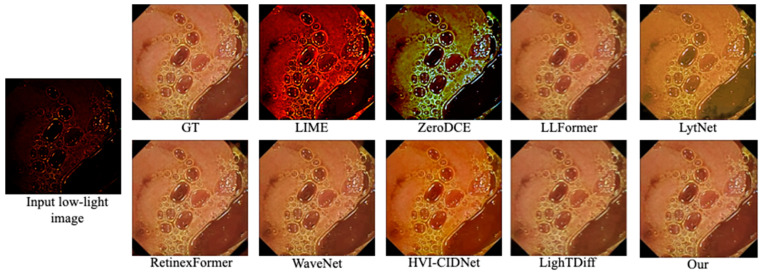
Visual comparison on the Kvasir-Capsule dataset. GT is the ground truth. Our method preserves natural color balance and detail better than competing approaches.

Figure 2 shows a representative example from the KC dataset. Methods such as LIME and ZeroDCE often produce heavily over-saturated or reddish outputs, losing natural color fidelity. LytNet and HVI-CIDNet reduce darkness but struggle to reproduce accurate color tones, often making the mucosal regions appear washed out or overly yellow.

RetinexFormer and WaveNet handle illumination well but introduce slight artifacts (e.g., in the top-right corner), where subtle textures become distorted or “smoothed over”.

By contrast, our method balances the global and local illumination, yielding a more natural tone. Crucial structures, including small vessels around the perimeter, remain clear, with minimal color distortion.

We next evaluate the RLE dataset. As shown in Table 2, our method again outperforms existing models, achieving a PSNR of 34.030 dB, an SSIM of 0.936, and an LPIPS of 0.055. WaveNet yields the second-best performance with 31.405 dB in PSNR and 0.922 in SSIM, but its LPIPS remains higher (0.088), indicating more perceptual discrepancy. Traditional methods (LIME, ZeroDCE) again have very low PSNR and SSIM, demonstrating their limited applicability to challenging WCE images.

**Figure 3:**
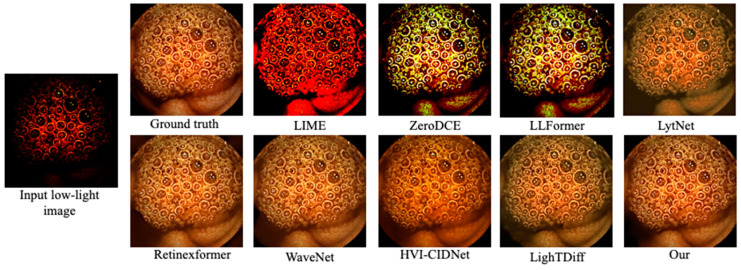
Visual comparison on the RLE dataset. Ground truth (top-left) vs. various enhancement methods. Our framework balances brightness and color fidelity, aiding in lesion visibility.

Figure 3 highlights visual comparisons of RLE images containing dense red lesions. LIME and ZeroDCE inadvertently produce strong color shifts (often too dark or overly bright). LLFormer, LytNet, and HVI-CIDNet show fewer color artifacts but still lose some micro-patterns in the darker regions. LightTDiff can yield pleasing global brightness but introduces mild color oversaturation. Our approach maintains the original hue of the mucosal tissues more faithfully while enhancing the visibility of the red lesion patterns.

Table 3 lists the no-reference image quality metrics PIQE and NIQE on an extended version of the Kvasir-Capsule dataset. While some approaches (e.g., LightTDiff with NIQE of 15.462) show low NIQE—indicating fewer “perceived artifacts”—their color shifts can still degrade medical detail. Our method’s NIQE (21.011) is higher, which often suggests a sharper image that the NIQE metric interprets as less “natural”. However, in clinical settings, sharper images can be preferable when evaluating subtle tissues. Nonetheless, these no-reference metrics must be interpreted cautiously, as they do not consistently reflect domain-specific requirements for medical imaging. Overall, the high-fidelity color and contrast delivered by our quaternion framework prove beneficial in medically relevant tasks, even if certain generic perceptual metrics do not fully capture these domain-specific advantages.

To further evaluate clinical relevance, we test the enhanced images on a red lesion segmentation task from the RLE dataset using TransUNet, a hybrid CNN-Transformer model known for capturing both global and local information [50]. TransUNet fuses the U-Net architecture with transformer encoders. A CNN backbone extracts high-resolution features, which are then fed into a transformer for global context modeling. The decoder merges multi-scale features via skip connections, similar to U-Net, enabling precise boundary localization of lesions.

Table 4 reports f1, mIoU, accuracy, precision, sensitivity, and specificity for red lesion segmentation under different enhancement outputs. Here, accuracy refers to standard pixel-wise accuracy: the fraction of correctly classified pixels over all pixels. The top row “NO (i.e., normal lighting) represents segmentation on normal, well-lit images, effectively serving as an upper bound. We then simulate low-light conditions and enhance them with each competing method before feeding into TransUNet.

LIME yields a large drop in segmentation metrics (f1 = 0.5713, mIoU = 0.3999) due to heavy over-saturation that confuses the lesion boundaries. ZeroDCE improves slightly but still struggles (f1 = 0.6061). LLFormer (f1 = 0.7715) and LytNet (f1 = 0.7507) perform better thanks to advanced low-light modeling. WaveNet (f1 = 0.7754) and HVI-CIDNet (f1 = 0.7768) further reduce over-smoothing and yield moderate gains. RetinexFormer (f1 = 0.7577) and LightTDiff (f1 = 0.7755) remain competitive.

Our method achieves the highest F1 score (0.7786) and maintains specificity (0.2792) close to the “NO” baseline. This indicates that our enhanced images allow TransUNet to recover subtle lesion boundaries, nearly matching its performance on well-lit data.

Although none of the methods fully replicate the “NO” condition, our quaternion Retinex approach comes the closest. This emphasizes the importance of color fidelity and preservation of local detail for effective medical image segmentation in dark or unevenly illuminated environments.

### 4.5. Ablation Studies

We conduct ablation experiments on the Kvasir-Capsule dataset to illustrate the importance of each key component—quaternion representation, wavelet-based decomposition, and the frequency-based loss term. First, We replaced our quaternion-based formulation with a standard RGB channel-wise approach (w/o Quaternion in Table 5). PSNR dropped by approximately 1.8 dB, and SSIM declined by 0.016, indicating that processing color channels independently reduces color consistency and overall image quality. Then, we trained the network entirely in the spatial domain (w/o Wavelet in Table 5). Removing wavelet decomposition caused a 1.2 dB decrease in PSNR and a 0.006 drop in SSIM. LPIPS also increased, showing that multi-scale frequency information aids both denoising and structure preservation. In the third experiment, we omitted the $L_{fft}$ term from the total loss. The visual enhancements remained plausible, but color banding and uneven illumination were more frequent. PSNR and SSIM decreased only slightly; however, LPIPS rose from 0.022 to 0.030, reflecting a noticeable drop in perceptual image quality.

The quaternion representation is important for preserving cross-channel color relationships, the wavelet transform substantially aids multi-scale illumination handling, and the FFT loss helps remove frequency artifacts for smoother illumination transitions.

These findings confirm that each design choice in QRNet contributes significantly to its performance on real low-light endoscopic images.

## 5. Discussion

This study presents a novel quaternion-based Retinex framework QRNet designed to tackle low-light and color distortion issues in endoscopic imaging. By concentrating on the unique requirements of capsule endoscopy—specifically, limited illumination, the need to maintain fine vessel and tissue details, and the importance of accurate color reproduction—our approach enhances state-of-the-art image enhancement and clinical utility (e.g., lesion segmentation). Below, we discuss the key aspects of our findings, potential limitations, and avenues for further exploration.

### 5.1. Effectiveness and Contribution

The proposed image decomposition framework effectively separates an image into multiple layers, each capturing distinct features (e.g., structural details, noise, illumination). This approach facilitates more targeted refinement, emphasizing subtle vascular patterns and mucosal textures while maintaining overall color consistency. In contrast, many existing methods depend on uniform adjustments across all channels and regions, often resulting in oversaturation or the loss of critical medical details. Our comparative analyses indicate that conventional algorithms, originally designed for general low-light photography, struggle to preserve fine structures in endoscopic images, frequently blurring vessel edges or causing significant color shifts.

By contrast, our quaternion-based Retinex design mitigates these artifacts. The proposed decomposition scheme helps in accurately extracting illumination and reflectance components, while the Quaternion Wavelet Attention (QWA) module refines crucial features more effectively. As our experiments on both the Kvasir-Capsule and RLE datasets demonstrate, this strategy yields higher PSNR, SSIM, and lower LPIPS compared to competing methods, underlining its ability to improve visibility in dim regions, preserve tissue color fidelity, and reduce noise. Furthermore, the enhanced images proved beneficial for downstream segmentation tasks. When evaluated with TransUNet, our approach provided f1 and mIoU values that closely approach those of well-lit “ground-truth” data, highlighting its potential to support improved diagnostic outcomes.

### 5.2. Limitations and Future Directions

While our method outperforms existing solutions, certain challenges and limitations warrant attention:

Dataset Diversity: Our experiments mainly depend on established datasets (Kvasir-Capsule, RLE). While these datasets contain clinically relevant images, they fail to adequately represent more extreme real-world conditions, such as highly occluded views or very low-light scenarios. Future efforts might involve acquiring specialized, rare, or exceptionally complex cases to further validate the algorithm’s robustness and generalizability.

Parameter Tuning: Certain hyperparameters (e.g., wavelet decomposition levels or Retinex-decomposition parameters) may need tailored settings for highly specific clinical contexts. Further research into adaptive parameter selection or automated hyperparameter search could enhance performance and streamline application in varied endoscopic procedures.

Broader Modalities: The conceptual Retinex-inspired design can transfer beyond capsule endoscopy to other medical imaging modalities—such as colonoscopy, bronchoscopy, or even fundus images—where consistent color fidelity and detailed vessel preservation are crucial. Exploring these domains could determine whether similar decomposition strategies yield comparable benefits in improving clinical diagnoses.

Boundary Handling: Like many enhancement methods, ours may have a diminished effect in regions where boundaries are poorly defined or heavily obscured. In future work, advanced multi-scale techniques or refined diffusion mechanisms might be explored to strengthen boundary reconstruction further.

Developments in image decomposition and attention mechanisms continue to show promise in medical image processing. Our findings suggest that incorporating color-aware operations, like quaternion color representation and Hamilton product, can substantially aid in recovering critical structures, especially under suboptimal lighting.

### 5.3. Relevance of Design Choices to WCE-Specific Enhancement Challenges

In this subsection, we discuss how each design choice in QRNet aligns with the requirements of WCE.

The unique challenges of Wireless Capsule Endoscopy (WCE) require carefully tailored solutions that address specific constraints and requirements. Our design choices in QRNet directly respond to these challenges through an integrated framework of complementary components.

The quaternion representation is a fundamental solution to WCE’s color preservation needs. In WCE imaging, subtle color variations are critical diagnostic indicators—from the deep red of active bleeding to the pale yellow of inflammation or the brown-black of melanomas. By encoding RGB channels as a unified quaternion entity rather than processing them independently, QRNet preserves the delicate inter-channel relationships that define these crucial color signatures. This approach has proven particularly effective in maintaining color fidelity even under extreme lighting conditions, where traditional channel-wise processing often introduces color distortions that could lead to misdiagnosis.

Our Retinex-based decomposition addresses one of WCE’s most significant challenges: the highly variable illumination caused by battery-powered LED light sources. WCE images typically display strong brightness gradients and shadows due to the capsule’s limited lighting capability and its constantly changing orientation within the GI tract. By separating images into reflectance and illumination components, QRNet optimizes each aspect independently, preserving critical tissue texture in the reflectance map while correcting uneven lighting in the illumination map. This separation is crucial for maintaining the visibility of mucosal patterns and vascular structures under varying lighting conditions.

The wavelet transform and attention mechanism work together to address WCE’s requirements for resolution and detail preservation. The wavelet decomposition effectively separates the image into multiple frequency bands, allowing the network to handle large-scale illumination changes and fine tissue details independently. This multi-scale approach is especially valuable in WCE, where broad lighting patterns and minute tissue features are clinically significant. The Quaternion Wavelet Attention (QWA) mechanism then selectively enhances important structural elements like vessel boundaries and mucosal patterns while suppressing noise. It is a critical capability, given WCE’s limited resolution and inherent image noise.

The frequency-based loss term specifically targets the global consistency challenges in WCE enhancement. Because the capsule moves and rotates, adjacent frames can exhibit significant variations in lighting and color. Our frequency-domain constraint ensures smooth, natural transitions in illumination and color across the image, preventing artificial boundaries or color banding that could interfere with interpretation. This global consistency is essential for accurately assessing extensive conditions such as inflammatory bowel disease or ongoing bleeding episodes.

These integrated design choices create a framework optimized for WCE’s unique imaging environment. The combination of quaternion processing, Retinex decomposition, wavelet-based attention, and frequency constraints enables QRNet to produce enhanced images that maintain diagnostic accuracy while overcoming WCE’s inherent limitations in lighting, color fidelity, and image quality. This comprehensive approach ensures that enhanced images serve their clinical purpose without introducing artifacts or distortions that could compromise diagnostic accuracy.

## 6. Conclusions

This paper presents a pioneering quaternion-based Retinex framework that significantly enhances wireless capsule endoscopy (WCE) image. By leveraging quaternion QRNet convolution layers and wavelet attention mechanisms, our approach performs better in preserving color fidelity, enhancing image quality, and addressing critical challenges in endoscopic imaging. Key achievements include the following:

- Quantitative improvements across standard metrics (PSNR: +2.3 dB, SSIM: +0.089, LPIPS: −0.126);
- A 5% enhancement in lesion segmentation accuracy;
- Preservation of critical inter-channel color relationships;
- Robust performance in challenging lighting conditions;
- Effective balance between enhancement and diagnostic fidelity;

Validation of the Kvasir-Capsule (2000 images) and Red Lesion Endoscopy (1500 images) datasets demonstrates QRNet’s effectiveness in real-world scenarios. The framework’s ability to maintain color fidelity while improving subtle tissue structure visibility directly addresses key limitations in current WCE technology. This work represents a significant step toward improving the early detection of GI abnormalities through enhanced WCE imaging quality. The demonstrated improvements in image quality and diagnostic accuracy position QRNet as a valuable tool for clinical practice, potentially reducing physician review time and improving patient outcomes.

Future research directions include Expanding the framework to extreme low-light conditions and occluded scenarios, investigating real-time processing capabilities for live endoscopic procedures, integrating automated quality assessment metrics for clinical deployment, and expanding validation to diverse pathological conditions and tissue types.

## Figures and Tables

**Table 1:** Results on the Kvasir-Capsule dataset. Bold numbers indicate the best performance for each metric, and underlined numbers indicate the second-best result.

Method	PSNR	SSIM	LPIPS
LIME [8]	12.058	0.3021	0.460
ZeroDCE [47]	13.671	0.446	0.515
LLFormer [48]	34.419	0.962	0.032
LytNet [31]	30.302	0.948	0.071
WaveNet [49]	35.893	0.975	0.026
HVI-CIDNet [32]	21.595	0.798	0.102
RetinexFormer [27]	33.660	0.959	0.033
LighTDiff [7]	31.413	0.920	0.067
QRNet (ours)	**37.781**	**0.976**	**0.022**

**Table 2:** Results on RLE dataset. Bold numbers indicate the best performance for each metric, and underlined numbers indicate the second-best result.

Method	PSNR	SSIM	LPIPS
LIME [8]	14.248	0.225	0.513
ZeroDCE [47]	15.277	0.323	0.429
LLFormer [48]	30.021	0.898	0.094
LytNet [31]	25.556	0.823	0.189
WaveNet [49]	31.405	0.922	0.088
HVI-CIDNet [32]	24.083	0.702	0.140
RetinexFormer [27]	28.724	0.891	0.097
LighTDiff [7]	31.413	0.920	0.067
QRNet (ours)	**34.030**	**0.936**	**0.055**

**Table 3:** Kvasir-Capsule results with perceptual metrics. Lower is better for NIQE, though these metrics can misalign with medical imaging priorities. Bold number indicates the best performance for each metric.

Method	NIQE
LIME [8]	46.345
ZeroDCE [47]	38.057
LLFormer [48]	31.432
LytNet [31]	50.041
WaveNet [49]	37.773
HVI-CIDNet [32]	43.748
RetinexFormer [27]	48.237
LighTDiff [7]	**15.462**

**Table 4:** Segmentation using TransUNet model. Bold numbers indicate the best performance for each metric, and underlined numbers indicate the second-best result.

Method	f1	mIoU	Accuracy	Precision	Sensitivity	Specificity
NO	0.7830	0.6434	0.6930	0.6587	0.9651	0.3266
LIME [8]	0.5713	0.3999	0.4775	0.5398	0.6068	0.3033
ZeroDCE [47]	0.6061	0.4349	0.5214	0.5743	0.6417	**0.3594**
LLFormer [48]	0.7715	0.6280	0.6718	0.6424	0.9656	0.2761
LytNet [31]	0.7507	0.6009	0.6316	0.6136	0.9666	0.1804
WaveNet [49]	0.7754	0.6332	0.6768	0.6449	0.9721	0.2792
HVI-CIDNet [32]	0.7768	0.6351	0.6825	0.6510	0.9629	0.3048
RetinexFormer [27]	0.7577	0.6100	0.6500	0.6285	0.9538	0.2407
LighTDiff [7]	0.7755	0.6333	0.6790	0.6478	0.9659	0.2927
Ours	**0.7786**	**0.6345**	**0.6919**	**0.6516**	**0.9725**	0.2792

**Table 5:** Ablation studies for QRNet. Bold numbers indicate the best performance.

Method	PSNR	SSIM	LPIPS
Full QRNet	**37.78**	**0.976**	**0.022**
w/o Quaternion	35.99	0.960	0.031
w/o Wavelet	36.56	0.970	0.027
w/o FFT Loss	37.44	0.975	0.030

## Data Availability

Publicly available datasets were used in this study. The synthetic low-light images and segmentation masks can be found here: longbai1006/LLCaps. The Kvasir-Capsule dataset can be found here: https://osf.io/dv2ag/ (accessed on 25 January 2025).
